# A practical workflow for applying validation strategies in tree height–diameter modelling^☆^

**DOI:** 10.1016/j.mex.2026.103982

**Published:** 2026-06-01

**Authors:** Santosh Ayer, Kishor Prasad Bhatta

**Affiliations:** a Faculty of Agricultural, Life & Environmental Sciences, University of Alberta, Edmonton T6G2E3, Canada; b Faculty of Forest Sciences and Forest Ecology, Georg-August-Universität, 37077 Göttingen, Germany

**Keywords:** Allometric models, Forest management, Height–diameter model, Model validation

## Abstract

Accurate validation of height–diameter (H–D) models is important for forest modelling applications, yet validation approaches are often applied inconsistently across forestry studies. This may produce different estimates of model performance depending on data structure and validation conditions. Therefore, the objective of this study was to present a practical workflow for applying internal, grouped, and external validation approaches in H–D modelling. Leave-one-out cross-validation (LOOCV), K-fold cross-validation, leave-one-district-out (LODO), leave-one-species-out (LOSO), and independent external validation strategies were included. Publicly available forest inventory datasets from Nepal were used to demonstrate the workflow, including a national multi-species dataset and two independent *Shorea robusta* datasets from western and far-western Nepal. The workflow demonstrated how different testing structures influence estimated model performance under within-dataset, grouped, and independent prediction conditions. This method provides a reproducible framework for validation in forest inventory and allometric modelling studies.

• A structured workflow is developed to select appropriate validation strategies based on data availability and modelling objectives.
• Internal, structured, and external validation are applied to assess model performance under different scenarios.
• A framework for the practical interpretation of validation results is provided to guide their use in general and site-specific modelling contexts.

## Graphical abstract

**Figure:**
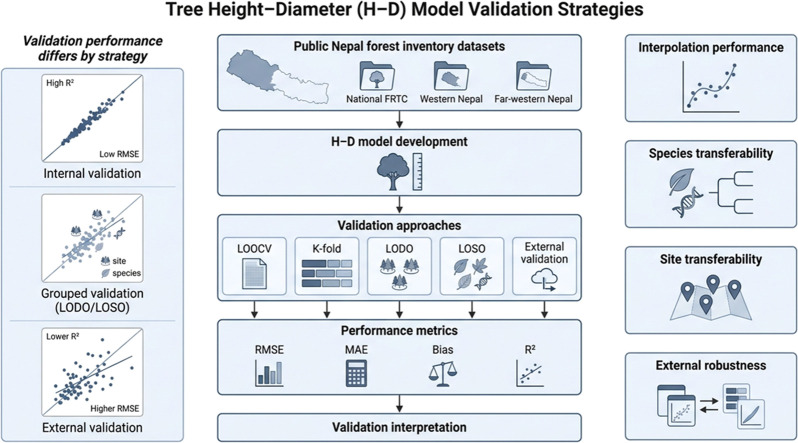

**Specifications table**

**Table:** 

**Subject area**	Agricultural and Biological Sciences
**More specific subject area**	Forest modelling and model validation
**Name of your method**	Workflow for selecting and interpreting validation strategies in height–diameter modelling
**Name and reference of original method**	This workflow integrates several commonly used validation strategies in forest allometric and ecological modelling studies. General cross-validation approaches including leave-one-out cross-validation (LOOCV) and K-fold cross-validation were informed by Arlot and Celisse [1]. Structured and grouped validation approaches, including leave-one-group-out validation, were informed by Roberts et al. [2] and Yates et al. [3], which discuss validation strategies under spatial, hierarchical, and structured data conditions. The application and interpretation of validation approaches in forest allometric modelling were informed by Paul et al. [4]. Independent external validation using datasets not involved in model fitting is widely recommended for evaluating model transferability under new site conditions
**Resource availability**	The method can be implemented using standard statistical software. R code used for model fitting and validation is available upon request. All datasets used in this study are publicly available including a Western Nepal dataset [5] and a Far-western Nepal dataset [6], and the multi-species national forest allometric model dataset was extracted from tables in a published FRTC report [7].

## Background

Height–diameter (H–D) models are widely used in forest inventory, growth modelling, and biomass estimation, as they provide a practical means of predicting tree height from diameter measurements. Therefore, accurate model validation is essential to ensure reliable predictions, particularly when models are applied across different sites, species, or environmental conditions [8,9]. However, the application of validation strategies remains inconsistent in practice. This issue is particularly evident in data-limited developing countries, where forest inventory datasets often have limited sample sizes, incomplete representation across geographic regions or species ranges, and a lack of independent datasets for external validation.

In such contexts, internal validation methods such as leave-one-out cross-validation (LOOCV) and K-fold cross-validation are commonly applied due to their simplicity and applicability in data-limited situations [[10], [11], [12]]. While these approaches provide useful estimates of model performance within the training dataset, they primarily assess interpolation accuracy and may not fully represent model performance under new or independent conditions [1]. More structured validation approaches, such as group-based or spatial cross-validation (e.g., leave-one-group-out or leave-one-species-out), can provide additional insights into model generalization across sites and data groups, but their application is often constrained by dataset structure and is not routinely implemented [2].

External validation using independent datasets is widely recognized as the most reliable approach for evaluating model transferability [4,13]. However, such datasets are rarely available in forestry studies, leading to reliance on internal validation methods without a clear understanding of their limitations [7,11]. As a result, there is often ambiguity in how validation results should be interpreted and how appropriate validation strategies should be selected for different modelling objectives.

To address these challenges, this study presents a reproducible workflow for applying validation strategies in tree height–diameter modelling. The workflow combines internal validation approaches (e.g., leave-one-out and K-fold cross-validation), structured leave-one-group-out validation approaches including leave-one-district-out (LODO) and leave-one-species-out (LOSO), and independent external validation within a single framework. The workflow was demonstrated using publicly available multi-species and site-level datasets containing diameter at breast height (DBH) and total tree height measurements.

## Method details

### Overview of the workflow

This study proposes a reproducible workflow for selecting and interpreting validation strategies in tree height–diameter (H–D) modelling. The workflow integrates internal validation, structured validation, and external validation within a unified framework to evaluate model performance under different data conditions. All analyses were conducted using the R statistical environment version 4.4.1 (R [14]). The workflow is reproducible for datasets containing diameter at breast height (DBH) and total tree height measurements, particularly for the studied tree species and similar forest inventory conditions. Internal validation approaches such as LOOCV and K-fold cross-validation can be applied to general datasets with sufficient sample size, whereas grouped validation approaches such as LODO and LOSO require grouped structures such as multiple species, sites, or geographic regions. Because the FRTC dataset [7] represents a national-level multi-species dataset collected across different regions of Nepal, the workflow can also be applied across different sites within the habitat range of the studied species.

### Data preparation

Three datasets were used to demonstrate the workflow. A multi-species forest inventory dataset was used for model development, while two independent site-level datasets were used for external validation. All datasets are publicly available. Two site-level datasets were obtained from Figshare, including a Western Nepal dataset [5] and a Far-western Nepal dataset [6], while the multi-species national forest inventory dataset was extracted from a published report by Forest Research and Training Centre of Nepal [7]. The national-level FRTC dataset was selected because it contained multiple commercially important tree species collected across different districts and forest regions of Nepal, making it suitable for demonstrating grouped validation approaches such as leave-one-district-out (LODO) and leave-one-species-out (LOSO) validation. In contrast, the Western Nepal and Far-western Nepal datasets were not used during model development with the FRTC dataset and were therefore used as independent datasets for external validation. These datasets were selected because they were publicly available and contained compatible DBH and total tree height measurements for *Shorea robusta*.

The FRTC dataset [7] consisted of 476 individual trees representing seven commercial species (*Alnus nepalensis, Castanopsis* spp., *Lagerstroemia parviflora, Pinus roxburghii, Schima wallichii, Shorea robusta*, and *Terminalia alata*). The Western Nepal dataset [5] originated from a destructive sampling study conducted in natural *Shorea robusta* forests of Kapilbastu district in western Nepal and included DBH and total tree height measurements of 180 harvested trees representing diameter classes from 30 to 95 cm. Similarly, the Far-western Nepal dataset [6] originated from a destructive sampling study conducted across multiple sites in Kailali and Kanchanpur districts of the far-western Terai region of Nepal and included DBH and total tree height measurements of 81 *Shorea robusta* trees representing multiple diameter classes. For consistency, variables were standardized across datasets by renaming columns to a common format (DBH and H). Diameter at breast height (DBH) was expressed in centimetres and total tree height in metres.

Observations were checked for missing values prior to analysis, and none were identified. In general, missing values should be removed or handled appropriately before model fitting. It is also recommended to visually inspect the data (e.g., scatterplots of DBH versus height) to identify potential outliers or inconsistencies before analysis. Since this study focuses on model validation, DBH and height measurements were used as provided in the original datasets, and detailed field measurement procedures are not described here.

### Model development

A nonlinear asymptotic H–D model was fitted to the data (Eq. (1)) where H represents total tree height (m), DBH is diameter at breast height (cm), and a and b are model parameters. This asymptotic form was selected because it reflects the biological pattern of height growth approaching an upper limit with increasing diameter and has been widely used in height–diameter modelling studies. Model fitting was performed using nonlinear least squares estimation in R. Initial parameter values were set to a=20 and b=0.05, which provided stable convergence; however, users may adjust these values depending on the range and variability of their data.

(1) $$H=1.3+a\left(1-e^{-b\cdot DBH}\right)$$

### Validation workflow

Internal validation was conducted using leave-one-out cross-validation (LOOCV) and K-fold cross-validation. In LOOCV, each observation was excluded sequentially, and the model was refitted to the remaining data to predict the excluded observation. In K-fold cross-validation, the dataset was randomly divided into five subsets, where each subset was used once as validation data while the remaining subsets were used for training. A fixed random seed is recommended to ensure reproducibility.

Structured validation was implemented using group-based cross-validation [3]. Leave-one-district-out (LODO) validation was conducted by grouping the dataset according to district and excluding one group at a time during model fitting, with the excluded group used for validation. Leave-one-species-out (LOSO) validation was performed by excluding one species from model training and using it for validation. These approaches allow assessment of spatial generalization and, in the case of LOSO, species-level generalization in generalized multi-species models.. Users should ensure that grouping variables are correctly defined and that each group contains sufficient observations for model fitting.

External validation was conducted using independent datasets not used in model development. The fitted model was applied directly to these datasets without recalibration, providing an assessment of model performance under new site conditions. Whenever independent datasets are available, external validation is recommended as it provides the most realistic evaluation of model transferability.

### Performance metrics

Model performance was evaluated using root mean square error (RMSE), mean absolute error (MAE), bias, and the coefficient of determination (R²). These metrics were calculated consistently across all validation approaches to allow direct comparison. Predictions containing missing values should be excluded prior to calculation to avoid biased estimates.

### Implementation notes and practical considerations

During implementation, several practical considerations were identified. Nonlinear model fitting may fail to converge for certain subsets of data during cross-validation; therefore, model fitting was implemented using a try–catch approach to handle convergence failures. It is recommended to monitor convergence warnings and adjust starting values if necessary. Datasets with limited variability or small sample sizes may produce unstable validation results, particularly for structured validation approaches such as LODO and LOSO. In addition, differences in data sources may influence external validation results and should be considered when interpreting model performance.

### Method validation

Model validation results obtained from the application of the proposed workflow are summarized in Table 1. Internal validation methods, including LOOCV and K-fold cross-validation, produced similar performance estimates, indicating consistent model behaviour within the training dataset. Structured validation approaches, such as LODO and LOSO, showed modest increases in prediction error and bias, reflecting the challenges associated with spatial and species-level generalization. External validation using independent datasets showed greater variability in model performance across sites, particularly in terms of bias and explanatory power.

**Table 1:** Comparison of height–diameter model performance across different validation strategies, including internal (LOOCV and K-fold), structured (LODO and LOSO), and external validation using independent datasets from Far-western and Western Nepal.

Validation Type	Method	RMSE	MAE	Bias	R²
Internal	LOOCV	4.146	3.34	0.032	0.727
Internal	K-fold	4.139	3.335	0.033	0.728
Structured	LODO	4.168	3.359	0.029	0.724
Structured	LOSO (*Shorea robusta* excluded)	4.354	3.593	1.742	0.732
External	Farwest Nepal	4.212	3.409	2.504	0.554
External	Western Nepal	3.02	2.416	1.806	0.709

These results demonstrate that different validation strategies capture distinct aspects of model performance and highlight the importance of selecting appropriate validation approaches based on data structure and intended application. The proposed workflow provides a practical framework for interpreting these differences in a consistent and reproducible manner. Based on these findings, recommended validation strategies under different data conditions are summarized in Table 2.

**Table 2:** Recommended validation strategies for height–diameter modelling under different data conditions, illustrating how the proposed workflow can be applied in practice.

Data condition	Recommended validation approach	Purpose
Limited dataset, no grouping	LOOCV or K-fold	Estimate general model performance
Dataset with spatial groups (e.g., sites, districts)	LODO	Assess spatial generalization
Multi-species dataset	LOSO	Assess species-level transferability in generalized models
Independent dataset available	External validation	Evaluate real-world model performance
No external data available	Internal + structured validation	Approximate model performance

## Limitations

The proposed workflow relies on the availability of structured datasets for implementing group-based validation approaches such as leave-one-district-out (LODO) and leave-one-species-out (LOSO). In situations where grouping variables (e.g., species or site identifiers) are not available, these validation approaches cannot be applied. External validation requires independent datasets, which may not always be accessible, particularly in data-limited contexts.

In addition, the performance of validation strategies may depend on dataset characteristics such as sample size, variability, and representativeness. Small or homogeneous datasets may produce unstable or overly optimistic validation results. Differences in data sources may also influence external validation outcomes and should be considered when interpreting results. Finally, although the workflow is demonstrated using height–diameter modelling, its application to other modelling contexts may require further evaluation.

## Ethics statements

Not applicable

## CRediT author statement

Santosh Ayer (Conceptualization, Formal analysis, Writing—original draft, Writing—review & editing); Kishor Prasad Bhatta (Writing—review & editing)

## Funding

This research did not receive any specific grant from funding agencies in the public, commercial, or not-for-profit sectors.

## Artificial intelligence (AI) disclosure

Artificial intelligence (AI) tools based on large language models, specifically ChatGPT (GPT-5, OpenAI, San Francisco, USA), was used to assist with language editing and improving the clarity of the manuscript. All analyses, results, and scientific interpretations were developed by the authors. The authors take full responsibility for the content of this manuscript.

## Data availability

All datasets used in this study are publicly available. The Western Nepal dataset is available at Figshare (https://doi.org/10.6084/m9.figshare.31938342), and the Far-western Nepal dataset is available at Figshare (https://doi.org/10.6084/m9.figshare.25225511). The multi-species national allometric model dataset was extracted from tables in a published FRTC report [7]. No new data were generated in this study.

## Declaration of competing interest

The authors declare that they have no known competing financial interests or personal relationships that could have appeared to influence the work reported in this paper.
